# Effectiveness of a Minimal Intervention for Stress-related mental disorders with Sick leave (MISS); study protocol of a cluster randomised controlled trial in general practice [ISRCTN43779641]

**DOI:** 10.1186/1471-2458-6-124

**Published:** 2006-05-04

**Authors:** Ingrid M Bakker, Berend Terluin, Harm WJ van Marwijk, Chad M Gundy, Johannes H Smit, Willem van Mechelen, Wim AB Stalman

**Affiliations:** 1 EMGO Institute, VU University Medical Centre Amsterdam, The Netherlands; 2 Department of General Practice, VU University Medical Centre Amsterdam, The Netherlands; 3 Clinical Epidemiology and Bio statistics, VU University Medical Centre Amsterdam, The Netherlands; 4 Department of Psychiatry, VU University Medical Centre Amsterdam, The Netherlands; 5 Department of Public and Occupational Health, VU University Medical Centre Amsterdam, The Netherlands

## Abstract

**Background:**

The main aims of this paper are to describe the setting and design of a Minimal Intervention in general practice for Stress-related mental disorders in patients on Sick leave (MISS), as well as to ascertain the study complies with the requirements for a cluster randomised controlled trial (RCT). The potential adverse consequences of sick leave due to Stress-related Mental Disorders (SMDs) are extensive, but often not recognised. Since most people having SMDs with sick leave consult their general practitioner (GP) at an early stage, a tailored intervention given by GPs is justified. We provide a detailed description of the MISS; that is more accurate assessment, education, advice and monitoring to treat SMDs in patients on sick leave. Our hypothesis is that the MISS will be more effective compared to the usual care, in reducing days of sick leave of these patients.

**Methods:**

The design is a pragmatic RCT. Randomisation is at the level of GPs. They received the MISS-training versus no training, in order to compare the MISS vs. usual care at patient level. Enrolment of patients took place after screening in the source population, that comprised 20–60 year old primary care attendees. Inclusion criteria were: moderately elevated distress levels, having a paid job and sick leave for no longer than three months. There is a one year follow up. The primary outcome measure is lasting full return to work. Reduction of SMD- symptoms is one of the secondary outcome measures. Forty-six GPs and 433 patients agreed to participate.

**Discussion:**

In our study design, attention is given to the practical application of the requirements for a pragmatic trial. The results of this cluster RCT will add to the evidence about treatment options in general practice for SMDs in patients on sick leave, and might contribute to a new and appropriate guideline. These results will be available at the end of 2006.

## Background

### Stress-related mental disorders (SMDs)

Stress results from an imbalance between demands and resources [1]. It is a psychological, physiological and behavioural response by individuals when they perceive a lack of equilibrium between the demands placed upon them and their ability to meet those demands. This response, over a period of time, leads to ill-health [2] and it is important to respect the roles of personal, social, economic, occupational and physical health problems in determining and shaping this psychological disability [3]. Fatigue, tenseness, irritability, apathy, sleeping disorder, emotional instability, rumination and concentration- problems are examples of common symptoms related to stress and a failure to cope with demands while resources (i.e. the abilities to meet those demands) are not sufficient. In addition, some patients with persistent distress go on to develop a psychiatric disorder, notably major depression or anxiety disorder, based on specific vulnerabilities. Plus, persistent distress may give rise to somatic complaints and subsequent somatization [4]. It is clear and evidence- based to treat major psychiatric disorders with medication or counselling, whereas evidence- based interventions for the whole range of Stress-related Mental Disorders (SMDs) are still under development.

### SMDs and sick leave

The societal and financial costs of dysfunction in terms of (long term) sick leave due to SMDs are extensive. In the Netherlands, almost one million workers are entitled to disability benefits (9 percent of the working population), this prevalence is high compared with other countries [5]. About one third of the 9.407 billion Euros in 2004 of the disability benefits in the Netherlands was paid to persons with mental health problems [6]. Whereas only ten percent of those receiving disability benefits have an actual psychiatric disorder, ninety percent is due to what patients and care-providers consider to be SMDs [7]. Furthermore, the composition of the group of workers who receive disability benefit in the Netherlands is changing disquietingly: currently young female employees, mostly with mental health problems, constitute the majority of new cases [8]. Chronicity of SMDs with sick leave is growing, although several studies indicate that SMDs can be treated successfully if they are diagnosed and treated at an early stage[9]. However, for a long time the usual approach to SMDs with sick leave was the reverse: advice to take rest and not return to work before all complaints had disappeared. Last but not least, patients with SMDs being on sick leave definitely cannot be reduced to only an economic problem; of course much personal suffering is involved. Moreover, the value of work is undisputed and in cases of (prolonged) sick leave there is a risk of permanent loss of employment.

### General Practice treatment of SMDs in patients on sick leave

Nearly every employee contacts the GP at the beginning of the sick leave. Most patients having SMDs are managed in primary care, and not referred to specialised secondary care. And despite the fact that mental health problems are common in primary care, GP's may still find it difficult to diagnose and treat them, unless they have a high index of suspicion [10]. Due to the collaborative nature of the doctor-patient relationship in general practice, many GPs may be overly cautious to attribute symptoms to a psychosocial cause. Another issue is an adequate differential diagnosis; many patients in primary care have symptoms related to anxiety, depression, somatization, or all three. In one out of five patients having a SMD there are prominent, although unrecognised symptoms of depression or anxiety, which are associated with a poor prognosis. A further pitfall is that GPs often tend to go along with the patient's request for rest and being left alone. Often GPs advise to go or stay on sick leave, to take rest, seek distraction and relaxation, instead of actively confront and cope with the difficulties. The distress of a lasting crisis combined with existing vulnerabilities may lead to prolonged disability and contribute to the development of serious mental disorders, e.g. depression or anxiety disorders. Earlier research revealed that about 20% of patients having a SMD stayed on sick leave for more than a whole year [11]. Cooperation between GPs and the occupational health care system seems to be in the best interest of all involved and also the preferred way to manage SMDs with sick leave.

### The Minimal Intervention for Stress-related mental disorders with Sick leave (MISS)

Terluin and Van der Klink [12] have outlined an activating intervention for patients having a SMD with sick leave, that already proved to be effective in reducing sick leave by 30% in an occupational health care setting [13]. Taken into account that GPs only have limited time during consulting hours, we developed the Minimal Intervention for Stress-related mental disorders with Sick leave (MISS) for general practice. In the MISS the principle of time contingency is used. Also, parts of more specialised psychological treatments like Cognitive Behavioural Therapy (CBT[14]) and Problem Solving Treatment (PST [15,16]) are incorporated. With respect to the role of gatekeeper in primary care practice, only basic principles of CBT and PST were considered relevant for GPs [17].

The MISS is a prototypical intervention for SMDs with sick leave in general practices, aiming specifically at successful rehabilitation and preventing long-lasting sick leave. By using specific communication and the minimal amount of time necessary, the GP helps the patient on the accurate and time-contingent course. The MISS should take no more than 3 consultations of 10–20 minutes, and consists basically of 5 elements: assessment, education, advice, monitoring and, if necessary, referral.

Assessment implies in the first place to identify patients having a SMD and help them to acknowledge their distress. Second, the GP detects significant depression and anxiety, and propose management steps for these problems separately. The Four-Dimensional Symptom Questionnaire (4DSQ, [18]) is used in the MISS to quantify the level of distress, and to detect symptoms of depression and anxiety. Guidelines from the Dutch College of General Practitioners are available to support the diagnosis and treatment of possible depression and anxiety disorders [19,20]. When physical illness has not yet been ruled out, the patient may have some somatic diagnostic examinations done, next to filling in the 4DSQ.

Education aims at promoting the patient's understanding and acceptance of the cause of the breakdown. Information is given on the normal course after having a SMD with sick leave, in this the patient's own active role is emphasised. The GP also gives information about the role and function of the occupational physician in the health care system. When the diagnosis may not yet be definitive, although the suspicion of a SMD is present, the education is still provided in the first consultation in order not to lose precious time.

Advice is given on coping with the breakdown, making a start with solving the problems, and planning to gradually take up social functioning. The GP underlines the importance of a balance in taking rest and making an active approach towards the problems, like using a rumination session (that is writing down what specifically troubles the patient) one or two times a day for 30–45 minutes. The patient is also advised to schedule necessary activities of daily living, such as children's care and housekeeping. Furthermore the patient is recommended to visit his or her occupational physician without delay, and to explore ways after which it is possible to partially return to work, even when not all symptoms have disappeared.

Monitoring is about ensuring the patient is moving towards the accurate course. This implies a focus on the problems (and possible solutions) in stead of symptoms. The patient has to investigate the specific problems and consider different ways to cope. The switch in the patient's focus has often already occurred before the second consultation; however it should in any case have occurred after four weeks of sick leave.

Referral to specialised care comes into play when there is no evidence of progression after four weeks of sick leave. The GP should realize that the patient is at risk for prolonged sick leave and ultimately loss of employment. A more specialised treatment is necessary since the patient is not likely to benefit from more time off. The GP may refer the patient to a counsellor, social worker or a (cognitive behavioural) psychotherapist, ideally after consulting with the occupational physician.

## Methods

### Objective

The central aim of this pragmatic cluster randomised controlled trial (RCT) is to investigate the effectiveness of the MISS in general practice. Our hypothesis is that GPs who carry out the MISS will be more effective than GPs who perform usual care in reducing the number of days of sick leave, as well as in reducing symptoms of SMDs in patients. Usual care in general practice contains the guidelines on depression [19] and anxiety [20]. In the case of the MISS, GPs are trained in topic- specific knowledge on patients with SMDs on sick leave. Because GPs, who have learned to apply the MISS, cannot be expected to perform this intervention in some patients and treat others as they used to do prior to the training, randomisation at the level of individual patients was not feasible. To avoid possible contamination between the conditions, a cluster design randomising at the level of GPs was chosen. There will be a one year follow up on the patients to assess the outcome measures and factors involved in the process of sick leave and return to work. The study design, protocol and procedures were approved by the Medical Ethics Committee of the VU University Medical Centre.

### Participants

#### GPs

The recruitment of GPs was split up in four different rounds. We approached GPs in two different districts where the Department of General Practice of the VU University Medical Centre has some type of network positioned. A total of 46 GPs signed informed consent.

#### Patients

In order to recruit enough eligible patients, we made use of the computerised patient record system and approached the source population of patients (n = 22.740) by mail. The source population consisted of all primary care attendees (20–60 years) who visited consulting hours of the participating GPs. GP's excluded only patients with very severe psychiatric disorders (mania or psychosis), patients with terminal illness or an inadequate command of the Dutch language. The source population of attendees was asked only to respond when they met our criteria: moderately elevated distress level (measured with 3 questions of the 4DSQ distress scale, [18]), having paid work and being (partially) on sick leave for no longer than three months (see table 1).

**Figure 1:**
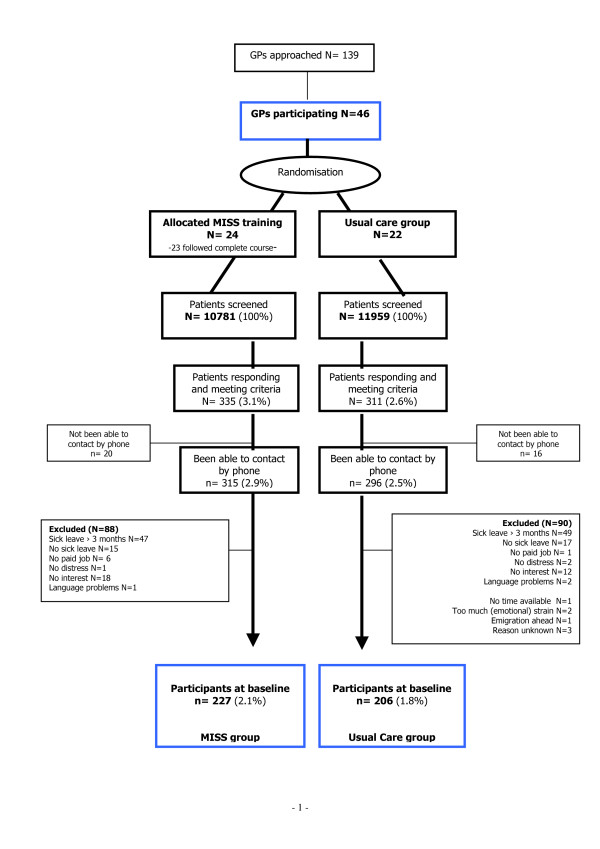
'Flow chart'.

Every one or two weeks we approached the source population, until enrolment of a sufficient number of patients from a particular GP was realised. Final recruitment took place by phone survey of the patients who returned the questionnaire and met the criteria. A total of 433 patients (1,9%) who could and also wanted to participate were enrolled (see figure 1). The overall response percentage on our screening method was 51.5%, this was measured in a group of 336 randomly selected attendees. This labour-intensive however highly successful method of screening ensures that we recruited patients having SMDs with sick leave in stead of patients who actually get an intervention for their complaints.

### Intervention at the level of general practitioners

In order to use the intervention, additional training in the MISS was given to intervention GPs by one of the authors (BT), and an occupational physician. This training existed of two times 3,5 hours and 2 times follow-up of 2 hours (total of 11 hours). Skills needed for successful treatment were accentuated. The participating GPs own experiences were evaluated; they were expected to provide case histories and to practice with the different parts of the intervention during the training. Screening of eligible patients started after the MISS group got the second training. When a patient had signed the informed consent (about four weeks after oral informed consent in the telephonic baseline measure), the GP was informed of the participation. Actual application of the elements of the MISS and steps in usual care are measured with a questionnaire.

### Intervention at the level of the patients

During the baseline interview all patients were asked whether they had planned another visit to their GP. If not they were advised to consider this, in order to give the GPs the opportunity to start with an intervention. Even though, it should be noted that neither the GPs were obliged to apply the MISS or any other intervention for mental disorders, nor were the patients obliged to go visit their GPs. No intervention was done on the actual completion or successfulness of the application of the MISS, the present method comprises real clinical practice in primary care.

### Outcome measures

#### Sick leave

The primary outcome measure is defined as: duration of sick leave in calendar days from the first day of sick leave to full return to work, lasting at least 4 weeks without (partial or full) relapse. Variables of the rehabilitation process itself, like the time to first (partial or full) return to work, total days of sick leave in the whole year, and (partial or full) return to work rates after 2, 6 and 12 months, are secondary outcome measures. Sick leave in the past year is considered to be a prognostic factor for our primary outcome measure, as are job content data [21,22].

#### Reduction of SMD symptoms

An important secondary outcome measure is reduction in symptoms of depression, anxiety, somatization and distress, measured with the 4DSQ [18]. Life-events and problems, chronic illnesses and neuroticism [23] are prognostic measures for this outcome. Problem evaluation [24] and coping styles [25] are measured to evaluate the effective components of the MISS: problem- and solution focus skills of patients.

#### Economic evaluation

Cost effectiveness will be evaluated from societal perspective (that is, irrespective of who is paying for the costs to gain an effect) by using the Tic-P [26]. The employers perspective (expenditures for the employer) is represented by the HPQ [27,28]. The EuroQol [29] measures general health state, and as a result quality of life status, that can be compared with a wide range of conditions in health care.

### Data collection

At baseline, patients who entered the study were measured by a phone survey and received a questionnaire by mail. Follow-up measurements, again a phone survey followed by a questionnaire, were scheduled at 2, 6 and 12 months after baseline. All outcomes were measured at the level of the patient, except for the GP interventions and skills. These were reported by the GPs two months after baseline. Moreover, data on received health care were extracted from the medical records after the completion of the one year follow-up.

### Power & sample size

Proportions used to determine the sample size needed, were adopted from a related study completed in the occupational health care setting [13]. In that study, after a period of 3 months 79% in the intervention group versus 64% in the control group had fully returned to work. In order to detect a relevant difference in survival analysis on our primary outcome measure, nQuery Advisor [30] was used to calculate the sample size. With a power of 80% at a 0.05 level two-sided log-rank test for equality of survival curves between the MISS group proportion still on sick leave of 0.21 and a usual care group proportion of 0.36 at the given time of 3 months, the sample size needed in each group was 126 (with a constant hazard ratio of 1.528). Taking into account an intracluster correlation coefficient (ICC) of .025 (clustering effect in our groups is not presumed to be large) for randomisation at GP level and 7 patients per cluster (GP), a total of 290 patients are needed. Assuming a dropout rate of 30% (approximately 10% at each follow up), inclusion of a total of 415 patients is necessary.

### Randomisation

Randomisation took place at the level of GPs, after each of the four recruitment moments and after the GPs signed informed consent. Because balance between groups in size and characteristics was presumed all four times GPs were randomised, no blocking or stratification was used. We developed the following procedure to conceal allocation. The names of the GP's (and dummy in an uneven group) were put on a list of which the order was subsequently randomised by one person (IMB). Another person (BT) independently drew up a randomised list of codes (1 = MISS group and 2 = control group) with an equal number of '1' and '2' codes up to the number of GPs being randomised. Finally, these two lists were brought together and the first GP on the list was allocated to the group indicated by the first code; and so on. As a result, 24 GPs were allocated to the intervention (MISS) group and 22 GPs were allocated to the control (usual care) group.

### Implementation

After assigning the GPs and training was given to the MISS group, patients were enrolled by screening the source population (see participants). The general practice team gave entrance to data on the source population, the source population was given the inclusion criteria through a screening questionnaire. The research assistance team was responsible for the final recruitment. They gave information in a phone survey and asked the patient informed consent to participate.

### Blinding

Patients were kept unaware that two different interventions were studied; both groups were given exactly the same information and questionnaires. The patients, as well as the external interviewers who carried out the phone surveys, were told that the study was about stress and sick leave. Finally, the internal research assistance team responsible for the process of data collection knew that the study involved a training of half of the GPs; nevertheless the internal research team had no information on which GPs were allocated to what conditions.

### Statistical methods

First of all, baseline similarity between the MISS and usual care groups will be examined, and baseline characteristics of drop outs and completers will be compared. Cox regression analyses will be used to investigate the intervention effect, by analysing differences in outcome with survival analysis of the primary outcome measure between the MISS and usual care group. To correct for misclassification of patients and severity of complaints (inclusion is only by level of distress with sick leave), and as a consequence to avoid bias in the effect, baseline measures of psychological symptoms (by means of the 4DSQ [31] & PRIME-MD [32]), as well as medical records will be examined. Additionally, subgroup analysis can be done by level of severity of complaints.

Linear and logistic multilevel analyses will be used to investigate the intervention effect on all secondary outcome measures: rates of return to work, psychological symptoms, problem experience and coping style. Also, longitudinal multivariate analysis will be used to examine differences in improvement in all secondary outcome measures between the treatment groups. Analysis will be performed on an intention-to-treat basis and intra-class-correlation will be calculated to correct for possible clustering of observations. Levels included will be: repeated measures, patients and GPs. Subgroups for analysis will be modelled by the prognostic factors mentioned.

Costs will be measured and valued from a societal perspective. Mean direct and indirect medical costs (measured with the Tic-P[26]), costs of productivity loss due to sick leave (measured with the WHO HPQ[28]) and total costs will be compared between both groups. Confidence intervals around mean differences will be estimated with bootstrapping methods. With regard tot the primary outcome, sick leave, a cost benefit analysis will be performed, in which costs of productivity loss due to sick leave will be compared with direct en indirect medical costs.

A cost-effectiveness analysis will be performed to assess the incremental costs per unit improvement on the 4DSQ [18]. Bootstrapping methods will be used to estimate the confidence interval for the cost-effectiveness ratio and to a draw cost-effectiveness plane. Similarly, utility assessed with the EuroQol [29] will be used to estimates the incremental costs per Qualy gained in a cost-utility analysis.

As regards the prognostic measures, univariate analyses will be used to select relevant factors, with a focus on identifying prognostic factors for our primary outcome measure. Subsequently, Cox regression analyses and logistic regression analysis will be performed on these relevant factors.

## Discussion

### SMDs in primary care

This project is developed for the primary care setting with its' typical case load of stress-related mental disorders, and not for specialised care in which patients have more clearly defined mental disorders. We provide an intervention that is aimed at better recognition, good communication, and a time-contingent framed recovery process. Our approach of SMDs, with the need to identify specific psychiatric disorders where they exist and also to respect the roles of daily life in determining and shaping psychological disability, is an example of specified stepped care in general practice. Potential risk factors for chronicity are pointed out in our training, and early recognition and treatment is the main goal of the MISS. The role of more specialised care is well acknowledged in the intervention.

### Benefits of our screening method

Particular strength of our study protocol is the method of recruiting patients. By using the computerised patient record system, we screened the whole population of general practice attendees and thereby determined who entered the study, instead of chartering the GPs to select their patients. Therefore, similarity in both groups is assured and we avoided selection bias. Also, we did not have difficulties in including enough eligible patients, which is often a problem in primary care trials. Furthermore, the current method allows us to consider the pragmatic effectiveness and to avoid interference with daily practice of consulting hours. In this trial, follow-up is on patients having SMDs with sick leave, instead of patients who actually get an intervention for their complaints. Good external validity (i.e. generalizability) is accomplished by this rather heterogeneous, and therefore highly representative, group of patients with SMDs on sick leave. To assure as much internal validity as possible in this pragmatic trial, we randomised on the level of GPs to avoid contamination. In addition, research assistants who collected the data and also the patients were blinded.

### Prospect on outcomes

Notably, effectiveness instead of efficacy is studied. We are evaluating what is possible in real clinical practice, rather than under ideal circumstances. As a consequence, mental health state will vary between the participants. Through subgroup analysis on severity of complaints and levels of distress (measured with the PRIME-MD and 4DSQ), we can classify possible high or low risk groups for prolonged disability within this heterogeneous group. Identification of a high risk group for non-recovery may lead to better suited guidelines on stepped care and treatment. We cannot assure that everyone in the MISS group has received the intervention; the GPs were given total freedom in actually delivering the MISS. Nevertheless, the number of visits, diagnoses, recommendations, treatments and proceedings will give us information about the compliance of the GPs and their influence on the effect of the MISS. In this way, daily practice is measured in stead of ideal circumstances. To avoid social desirable answers from the GPs on their advises and treatments, we will also check the medical records of the patients.

Finally, many requirements for a high quality trial are being met. Results of this cluster RCT will contribute to treatment options for patients having SMDs with sick leave in general practice, and might contribute to new and better suited guidelines and stepped care. Results will be available in the end of 2006.

## Competing interests

The author(s) declare that they have no competing interests.

## Authors' contributions

BT developed the intervention. BT and HvM participated in the design and co-ordination of the trial, and in writing the article. JS advised on the content of the design of the trial. CG advised on the content of statistics. WS and WvM advised on the content of the study design and article. IMB conducted the research and wrote the article. All authors provided comments on the drafts and have read and approved the final version.

## Pre-publication history

The pre-publication history for this paper can be accessed here:



## Figures and Tables

**Table 1:** Eligibility criteria

	No	Sometimes	Regularly or often
1. During the past week, did you suffer from worry?	0	1	2
2. During the past week, did you suffer from listlessness?	0	1	2
3. During the past week, did you feel tense?	0	1	2
			
4. Total score 4 or higher?	□ Yes	□ No	
5. Do you currently have a paid job?	□ Yes	□ No	
6. Are you currently on sick leave for a period no longer than three months?	□ Yes	□ No	

People were asked only to send in the questionnaire when they scored three times 'yes' and were willing to participate.

**Table 2:** Outcome measures

		**Baseline**	**Follow up in months**		
**Outcome measure**	**Instrument**	**0**	**2**	**6**	**12**

**Primary outcome (dependent)**					
Lasting return to work: duration of sick leave in calendar days from the first day of sick leave to full return to work, for at least 4 weeks without (partial or full) relapse	Patients report	X	X	X	X

**Secondary outcomes (dependent)**					
Time to first (full or partial) return to work	Patients report	X	X	X	X
Return to work rates	Patients report	X	X	X	X
Total days of sick leave during one year follow up	Patients report	X	X	X	X
Recurrence of sick leave	Patients report	X	X	X	X
Reduction of symptoms	4DSQ [18,31]	X	X	X	X
Health state profile	Euroqol [29]	X	X	X	X
Costs of health care and loss of productivity	Tic-P [26]	X	X	X	X
Absenteeism, quality and quantity of work	HPQ [27,28]	X		X	
Problem evaluation	Psychlops (MYMOP) [24,33]	X	X	X	X
Coping processes	Ways of Coping Questionnaire [25]	X	X	X	X
Patient satisfaction	Patients report		X	X	X
Application of the MISS: number of visits, diagnosis, advice & treatment, proceeding of recovery process over the past year	Medical record & questionnaire filled in by GP				X

**Prognostic measures (independent predictor or covariate)**					
Mental disorders	PRIME-MD [32,34]	X			
Sick leave in year before	Patients report	X			
Problems, life events, chronic illnesses	Patients report	X		X	X
Work experience/burn out	UBOS [21]	X			X
Job content data, job stress	JCQ [22]	X			
Critical incidents	HPQ [27]	X		X	
Neuroticism	NEO-FFI [23]	X			

## References

[B1] Lazarus RS, Folkman S (1984). Stress, appraisal and coping.

[B2] Palmer S (1989). Occupational Stress. The Health and Savety Practitioner.

[B3] Middleton H, Shaw I (2000). Distinguishing mental illness in primary care. BMJ.

[B4] Burton C (2003). Beyond somatisation: a review of the understanding and treatment of medically unexplained physical symptoms (MUPS). British Journal of General Practice.

[B5] OECD Economic Surveys (2004). Netherlands: Reform of the sickness and disability benefit schemes.

[B6] Workers Insurance Authority (UWV) (2005). Costs of disability benefits in 2004, defined by ICD-10 diagnoses.

[B7] Schreurs P, Taris T, Baldal M, Iping PJM, Zwaard AW (2001). Work pressure, work stress, psychological fatigue and burnout. Annual occupational and internal environment 2001.

[B8] Grünell M Young employees with psychological complaints add to growth in disability benefit claimants. european industrial relations observatory on-line.

[B9] Health Council of the Netherlands (Gezondheidsraad) (2005). Diagnoses, treatment, guidance. Medical interventions on sick leave and disablement. (only available in Dutch) 2005/10.

[B10] Hickie IB (1999). Primary care psychiatry is not specialist psychiatry in general practice. Medical Journal of Australia.

[B11] Schröer CAP (1993). Absenteeism due to 'overstrain'. A study of the nature of overstrain, therapeutic assitance and absenteeism [In Dutch, with a summary in English].

[B12] Klink JJLvd, Terluin B (2005). Psychological problems and work. Manual for an activating guidance by the general practitioner and occupational health worker [In Dutch].

[B13] van der Klink JJL, Blonk RWB, Schene AH, van Dijk FJH (2003). Reducing long term sickness absence by an activating intervention in adjustment disorders: a cluster randomised controlled design. Occupational and Environmental Medicine.

[B14] Beck JS (1995). Cognitive therapy: Basics and beyond.

[B15] D'Zurilla TJ, Goldfried MR (1971). Problem solving and behavior modification. Journal of Abnormal Psychology.

[B16] Mynors-Wallis L (1996). Problem-solving treatment: evidence for effectiveness and feasibility in primary care. International Journal of Psychiatry in Medicine.

[B17] Huibers MJH, Beurskens AJHM, Bleijenberg G, van Schayck CP (2003). The effectiveness of psychosocial interventions delivered by general practitioners (Cochrane Review). The Cochrane Library.

[B18] Terluin B, Van Rhenen W, Schaufeli WB, De Haan M (2004). The Four-Dimensional Symptom Questionnaire (4DSQ): measuring distress and other mental health problems in a working population. Work and Stress.

[B19] van Marwijk HWJ, Grundmeijer HGLM, Bijl D, van Gelderen MG, De Haan M, van Weel-Baumgarten EM, Burgers JS, Boukes FS, Romeijnders ACM (2003). Dutch College of General Practioners Guideline Depression, first revision [NHG-Standaard Depressieve stoornis (depressie). Eerste herziening. In Dutch]. Huisarts en Wetenschap.

[B20] Terluin B, Van Heest FB, Van der Meer K, Neomagus GJH, Hekman J, Aulbers LPJ, Starreveld JS, Grol MH (2004). Dutch College of General Practioners Guideline Anxiety disorder, first revision [First Revision. In Dutch]. Huisarts en Wetenschap.

[B21] Maslach C, Jackson SE, 'Utrecht Burnout Scale' by: Schaufeli WB, Van Dierendonck D (2000). MBI: Maslach Burnout InventoryManual (research edition).

[B22] Karasek RA (1979). Job Demands, Job Decision Latitude, and Mental Strain – Implications for Job Redesign. Administrative Science Quarterly.

[B23] Costa PT, McCrae RR, Hoekstra HA, Ormel J, Fruyt F de (1996) (1992). NEO PI-R personality inventory. NEO FFI five factor inventory.

[B24] Paterson C (1996). Measuring outcomes in primary care: A patient generated measure, MYMOP, compared with the SF-36 health survey. British Medical Journal.

[B25] Bramsen I, Bleiker EMA, Triemstra AHM, VanRossum SMG, vanderPloeg HM (1995). A Dutch adaptation of the ways of coping questionnaire: Factor structure and psychometric properties. Anxiety Stress and Coping.

[B26] Hakkaart van Roijen L (2002). Trimbos/iMTA questionnaire forCosts associated with Psychiatric Illness (Tic-P).

[B27] Kessler RC, Barber C, Beck A, Berglund P, Cleary PD, McKenas D, Pronk N, Simon G, Stang P (2003). The world health organization health and work performance questionnaire (HPQ). Journal of Occupational and Environmental Medicine.

[B28] Kessler RC, Ames M, Hymel PA, Loeppke R, McKenas DK, Richling DE, Stang PE, Ustun TB (2004). Using the World Health Organization Health and Work Performance Questionnaire (HPQ) to evaluate the indirect workplace costs of illness. Journal of Occupational and Environmental Medicine.

[B29] Kind P, Spilker B (1996). The Euroqol Instrument: an index of health-related quality of life. Quality of life and pharmaoeconomics in clinical trials.

[B30] (2005). nQuery Advisor^®^. http://www.statsol.ie/nquery/nquery.htm.

[B31] Terluin B (1994). 4DSQ. http://www.emgo.nl/researchtools/4dsq.asp.

[B32] Spitzer RL, Williams JBW, Kroenke K, Linzer M, Degruy FV, Hahn SR, Brody D, Johnson JG (1994). Utility of New Procedure for Diagnosing Mental-Disorders in Primary-Care – the Prime-Md-1000 Study. Jama-Journal of the American Medical Association.

[B33] Paterson C (2004). Seeking the patient's perspective: A qualitative assessment of EuroQol, COOP-WONCA charts and MYMOP. Quality of Life Research.

[B34] Spitzer RL, Kroenke K, Williams JBW (1999). Validation and utility of a self-report version of PRIME-MD – The PHQ primary care study. Jama-Journal of the American Medical Association.

